# Incidence rates and characteristics of abnormal lumbar findings and low back pain in child and adolescent weightlifter: A prospective three-year cohort study

**DOI:** 10.1371/journal.pone.0206125

**Published:** 2018-10-29

**Authors:** Kengo Shimozaki, Junsuke Nakase, Katsuhito Yoshioka, Yasushi Takata, Kazuki Asai, Katsuhiko Kitaoka, Hiroyuki Tsuchiya

**Affiliations:** 1 Department of Orthopedic Surgery, Graduate School of Medical Sciences, Kanazawa University, Takara-machi, Kanazawa-shi, Ishikawa-ken, Japan; 2 Department of Orthopedic Surgery, Kijima Hospital, Matsutera-machi, Kanazawa-shi, Ishikawa-ken, Japan; Medical College of Wisconsin, UNITED STATES

## Abstract

**Purpose:**

The purpose of this three-year cohort study was to assess the incidence rates and characteristics of abnormal lumbar findings and low back pain (LBP) in child and adolescent weightlifting athletes using magnetic resonance imaging (MRI) and medical questionnaires. This study evaluated subclinical sports injuries, which in turn may help prevent competition-specific injuries and improve performance levels.

**Materials and methods:**

Between 2014 and 2016, twelve participants who had been competing in weightlifting events for at least 2 years were enrolled in this study. The mean age of the participants at the start of this study was 11.4 ± 2.0 years, and there were 6 boys and 6 girls. Annual medical questionnaire surveys and lumbar examinations using MRI were performed during the 3-year follow-up. The incidence rates and variations of LBP and abnormal MRI findings were evaluated.

**Results:**

At the start of this study, there were no positive findings of LBP, and abnormal lumbar findings on MRI were observed in only 2 participants. At the 2-year follow-up, 1 participant had LBP, and 8 of 12 participants had abnormal lumbar findings. In the final year, only 3 participants had LBP; however, abnormal lumbar findings were observed on MRI in 11 participants. Among these, lumbar spondylolysis was observed in 4 participants, lumbar disc protrusion or extrusion in 2 participants, and lumbar disc degeneration in 9 participants.

**Conclusion:**

This prospective 3-year cohort study of 12 child and adolescent weightlifters revealed abnormal lumbar findings in 11 participants at a high rate on MRI examination. Children and adolescents undergoing resistance training at the competition level could potentially have irreversible changes in the lumbar vertebra without symptoms.

## Introduction

Resistance training activities such as weightlifting in children and adolescent athletes has been considered unsafe, as they may cause injuries to the musculoskeletal system, growth plate injuries, and low back pain [1–4]. Recently, many studies have reported that most of these sports injuries were caused by improper lifting techniques, poorly chosen training loads, or a lack of qualified adult supervision [5,6]. Milone et al. [7] reported that there is no need to avoid resistance training until epiphyseal closure. Additionally, it has been reported that resistance training when done under the supervision of a coach may lead to improved performance and prevent the occurrence of sports injuries [8–10]. It is becoming a trend to recommend well-designed training by medical, fitness, and sports organizations during this period.

Weightlifting causes more low back pain (LBP) than any other sport [11]. However, studies on the long-term implications of resistance training (weightlifting) in youth before the epiphyseal closure [7] are limited, and weightlifting at the competition level has not been recommended during the growth phase [12]. As such, the safety and validity of resistance training among children and adolescents, especially weightlifting at the competition level, has remained controversial.

The spontaneous incidence rate ofinjuries in children and adolescents during weightlifting has to be investigated to assess the short- and long-term risk of weightlifting at the competition level. Therefore, we focused on the lumbar vertebrae in our study, as this is most frequently injured region secondary to weightlifting [13], and prospectively investigated LBP and the abnormal lumbar findings in child and adolescent weightlifters.

The purpose of this our study was to assess the incidence and characteristics of LBP and abnormal lumbar findings in child and adolescent weightlifting athletes by using medical questionnaires and magnetic resonance imaging (MRI). This study was conducted to evaluate subclinical sports injuries, which in turn may help prevent competition-specific injuries and improve performance levels.

## Materials and methods

We conducted a prospective 3-year cohort study. Between 2014 and 2016, 12 participants who belonged to the weightlifting team we supported were enrolled in the study. The participants were either children or adolescents who continued to participate in weightlifting activities at competition levels for at least 2 years. They had no history of lumbar diseases or surgeries, metabolic diseases, or autoimmune diseases. This study was approved by the Ethical Committee of the Graduate School of Medical Sciences, Kanazawa University (approval #1399). Participants and their parents were given a thorough explanation of the study design and voluntarily provided written informed consent.The participants were followed up for 3 years. The mean age of the participants at the start of this study was 11.4 ± 2.0 years, and there were 6 boys and 6 girls. Annual medical questionnaires and lumbar examinations using MRI were conducted at the same season as part of medical support during the 3-year follow-up.

The assessment items included a medical questionnaire, which was used to record the practice frequency, presence of LBP at each year, and MRI findings. Subsequently, the annual changes of the practice frequency, LBP, and MRI findings were evaluated.

The definition of LBP for this study was that the participant was unable to practice weightlifting for more than a week due to the pain. Practice was stopped if the slightest pain was present and restarted when the pain runs out.

MRI of the lumbar vertebrae was performed with a flexible quadrature detection body coil on a 0.4 T unit (APERTO, Hitachi Medical, Tokyo, Japan). Each participant was fixed in the supine position with the knee joint in mild flexion. A T2-weighted image in the sagittal and coronal planes was used to evaluate the characteristic MRI findings. The section thicknesses of the coronal and sagittal views were 3.5 mm. The interval gaps for both views were 1.0 mm.

On MRI, the presence of lumbar spondylolysis, lumbar disc protrusion and extrusion, and lumbar disc degeneration were examined at all the lumbar vertebral levels (from L1 to S1) in the sagittal and coronal plane. Although computed tomography (CT) is regarded as the best tool for making an accurate diagnosis of lumbar spondylolysis [14], pedicle signal changes are better visualized on MRI rather than CT for detecting initial flesh lumbar spondylolysis in adolescents [15]. CT also has the problem of radiation exposure. For the purposes of this study, we defined pedicle signal changes as lumbar spondylolysis using MRI rather than CT.

**Fig 1:**
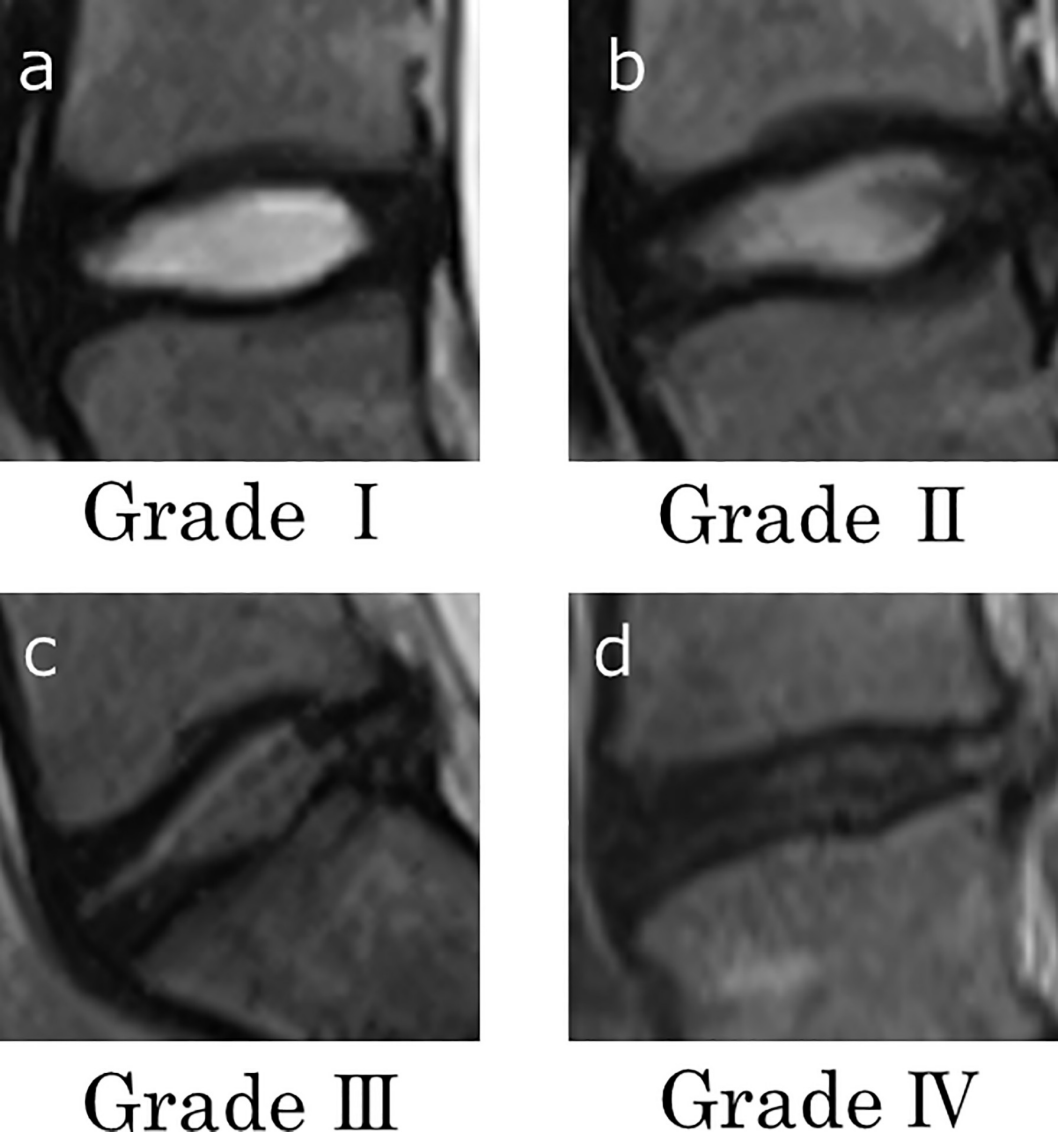
Standard T2-weighted MRI sagittal images of each Pfirrmann grade. We defined the Pfirrmann grade at each level of the lumbar vertebrae. Focused on the structure of the disc and disc height, it divided into grade **Ⅰ**(a), **Ⅱ**(b), **Ⅲ**(c), and **Ⅳ**(d), but grade **Ⅳ** was not absent in this study.

The Pfirrmann classification [16] was used for the assessment of lumbar disc degeneration (Fig 1, Table 1). MRI findings were interpreted by two orthopedic surgeons; a spine surgery specialist (K.Y) and an experienced orthopedic surgeon (K.S). Both specialists were unaware of the other findings of each participant. Each reader interpreted the MRI findings twice, with each time-point separated by a period of two weeks. When the judgments of the two surgeons differed, the two readers consulted and adopted the judgment of the spine surgeon. Inter-reader and intra-reader agreements were assessed using the κ value [17].

**Table 1:** Classification of intervertebral disc degeneration on magnetic resonance imaging (MRI) by Pfirrmann et al [16].

Grade	Appearance on T2-weighted sagittal images of spine
**Ⅰ**	The structure of the disc is homogeneous, with bright hyperintense white signal and a normal disc height.
**Ⅱ**	The structure of the disc is inhomogeneous, with bright hyperintense white signal. Disc height is normal, with or without a horizontal gray band.
**Ⅲ**	The structure of the disc is inhomogeneous, with an intermediate gray signal intensity. Disc height is normal or slightly decreased.
**Ⅳ**	The structure of the disc is inhomogeneous, with hypointense dark gray signal intensity. Disc height is normal or moderately decreased,

## Results

Under the guidance of a team coach, the participants practiced weightlifting for approximately 2 hours per day for an average of 5 days per week, averaging 500 hours per year. At the start of this study, there were no positive findings of LBP, lumbar spondylolysis, or disc protrusion and extrusion on MRI. Lumbar disc degeneration on MRI was observed in only 2 participants. At the 2-year follow-up, there was only 1 participant with LBP, and abnormal lumbar findings were observed in 8 of 12 participants. At the final measurement point in this study, abnormal lumbar findings were observed in 11 out of the 12 participants (91.7%), 1 (8.3%) had no abnormal findings on MRI, and 3 participants had LBP (Table 2). Among those with abnormal lumbar findings, lumbar spondylolysis was found in 4 participants, lumbar disc protrusion in 2 participants, and lumbar disc degeneration in 9 participants.

**Table 2:** Secular changes of lumbar spinal findings on MRI and symptoms over a 3-year period.

	2014	2015	2016
Lumbar disc degeneration (cases)	2	8	9
Lumbar spondylolysis (cases)	0	1	4
Lumbar disc protrusion and extrusion (cases)	0	0	2
Presence of low back pain (cases)	0	1	3

**Fig 2:**
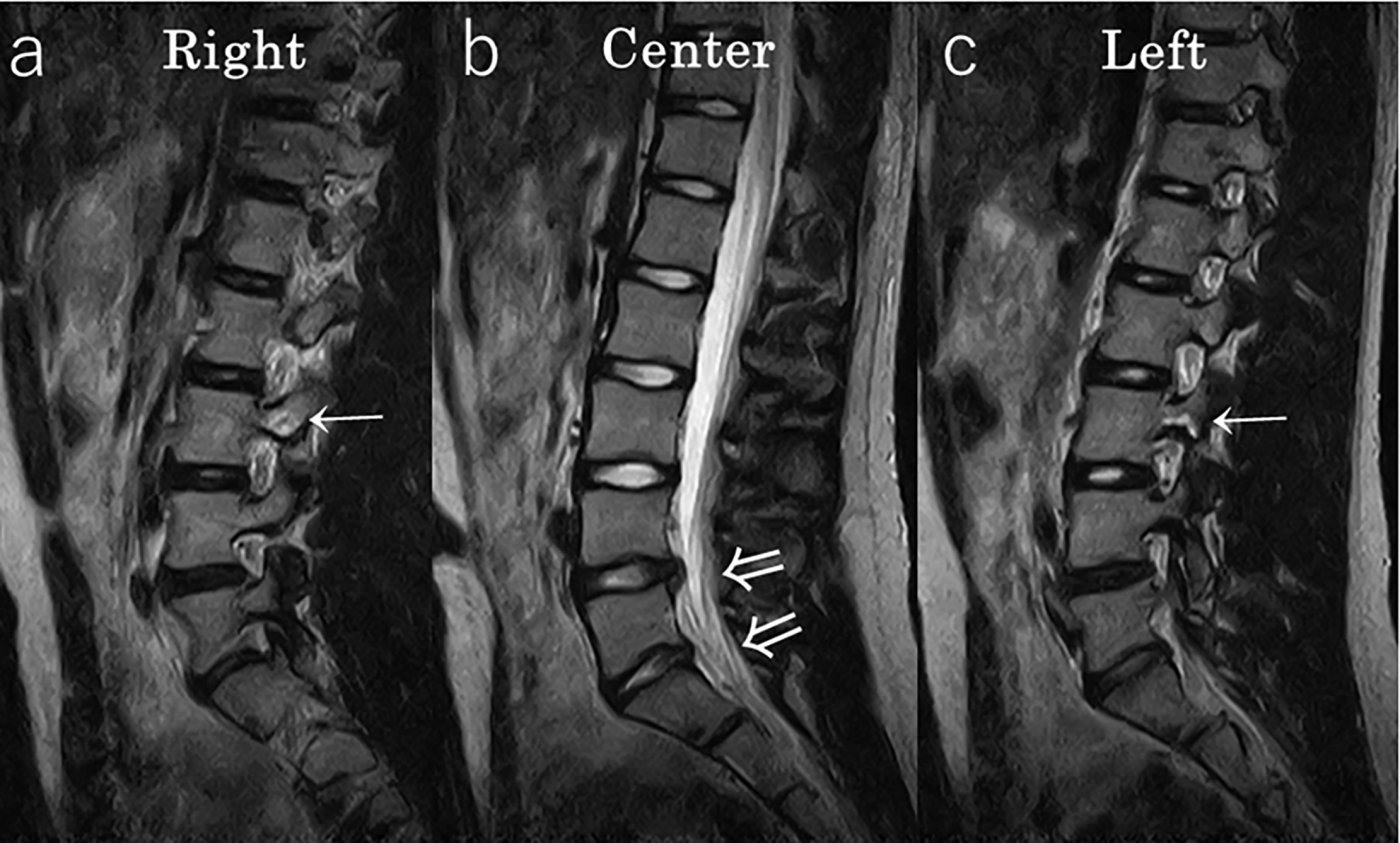
Representative case #1 MRI of a 14 year-old girl in 2016. Lumbar spondylolysis was noted bilaterally at the L3 level (a,c: →), disc protrusion was noted at the L4/5 level, and disc degeneration was found at the L4/5 and L5/S1 levels (b: ⇒).

**Fig 3:**
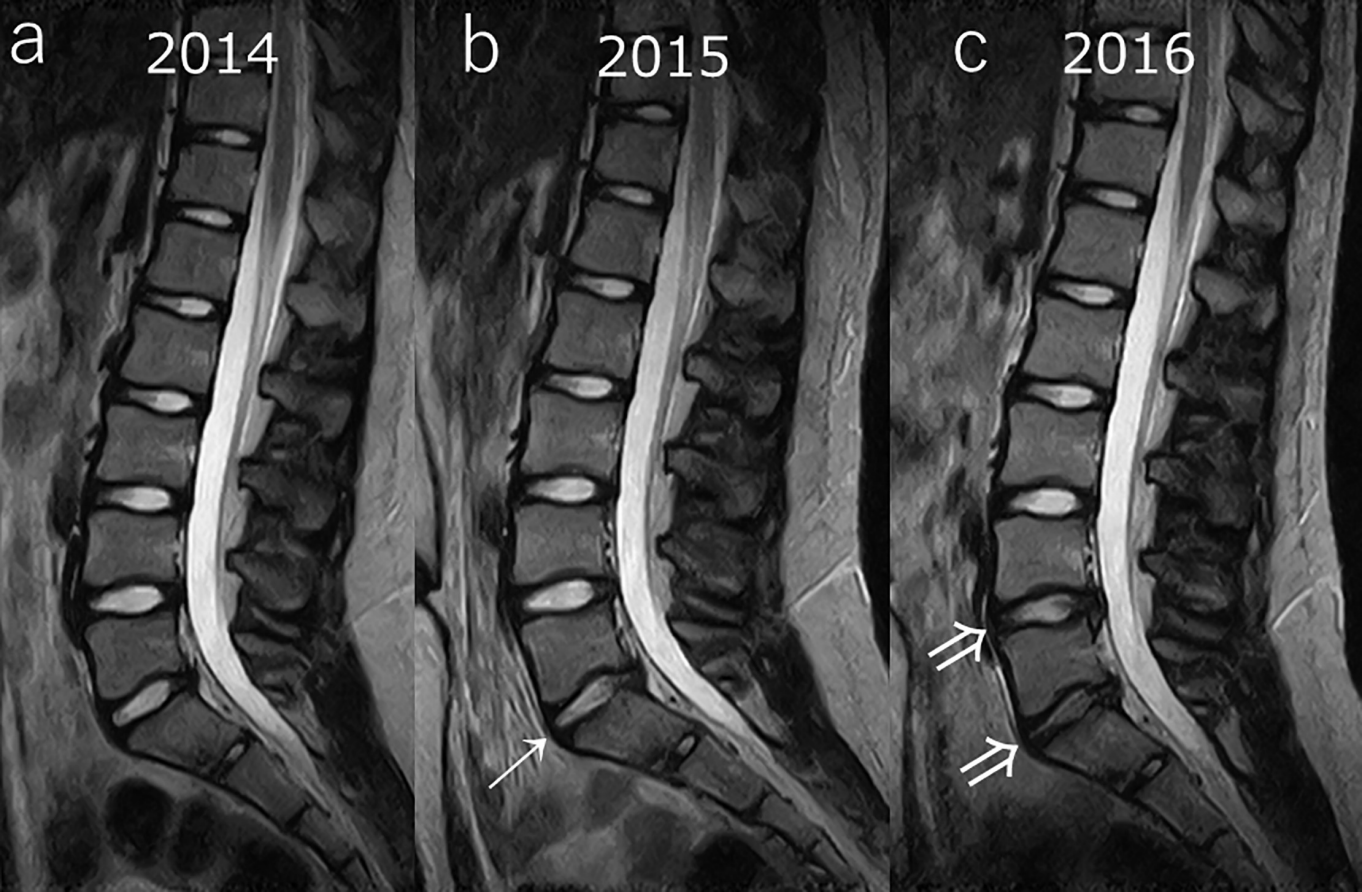
Representative case #2 MRI of a 12 year-old girl at the start of the study in 2014. There were no findings in 2014 (a); however, lumbar disc protrusion and disc degeneration were noted at the L5/S1 level in 2015 (b: →), and at the L4/5 and L5/S1 levels in 2016 (c: ⇒). The degenerative changes were irreversible at this point.

In the representative case shown in Fig 2, the participant presented with bilateral lumbar spondylolysis at the L3 vertebral level, disc protrusion at L4/5, and disc degeneration at L4/5, L5/S1 in the year 2016. Degenerative disc changes in the lumbar region were nearly irreversible. A representative case is shown in Fig 3, and the grades of the cases of degeneration between 2014 and 2016 are shown in Tables 3, 4 and 5. The κ value of inter-reader agreement was 0.78 (substantial), and intra-reader agreement was 0.68 (substantial, mean of the readers).

**Table 3:** The grade of lumbar disc degeneration at each lumbar vertebral level in 2014, as defined by the Pfirrmann classification*.

2014 Pfirrmann classification	Grade Ⅰ	Grade Ⅱ	Grade Ⅲ	Grade Ⅳ
L1/2	11	1	0	0
L2/3	12	0	0	0
L3/4	12	0	0	0
L4/5	11	1	0	0
L5/S1	12	0	0	0

*There were only 2 cases of Grade Ⅱ lumbar disc degeneration.

**Table 4:** The grade of lumbar disc degeneration at each lumbar vertebral level in 2015, as defined by the Pfirrmann classification*.

2015 Pfirrmann classification	Grade Ⅰ	Grade Ⅱ	Grade Ⅲ	Grade Ⅳ
L1/2	10	1	1	0
L2/3	10	2	0	0
L3/4	11	1	0	0
L4/5	6	4	2	0
L5/S1	10	2	0	0

*There degenerative changes were higher in 2015 than in 2014.

**Table 5:** The grade of lumbar disc degeneration at each lumbar vertebral level in 2016, as defined by the Pfirrmann classification*.

2016 Pfirrmann classification	Grade Ⅰ	Grade Ⅱ	Grade Ⅲ	Grade Ⅳ
L1/2	9	2	1	0
L2/3	10	2	0	0
L3/4	11	1	0	0
L4/5	4	6	2	0
L5/S1	10	1	1	0

*The lumbar disc degeneration was noted to be irreversible when compared to the findings in 2015.

## Discussion

In this 3-year prospective cohort study of child and adolescent weightlifting athletes, we found abnormal findings on MRI in 11 of the 12 cases (91.7%), despite the finding that 9 cases (75%) had no LBP.

The frequency of injury during weightlifting has been reported to be about 1.1–4.4 per 1000 hours, which is lower than in other sports [18,19]. Additionally, the incidence rate of injuries during weightlifting in children and adolescents can be further reduced by appropriate guidance in resistance training and weightlifting [5,9,20]. Faigenbaum et al. [21] evaluated the safety and efficacy of 1-repetition maximum training in 96 children (6- to 12-year-olds) and reported that maximal effort strength was acceptable for healthy boys and girls. However, in these studies, the evaluation of injuries was based on the symptoms of the athletes. There were no prospective studies focused on potential changes without any symptoms in athletes before epiphyseal closure.

As a result of this prospective study, evaluation of spontaneous symptomatic injuries and abnormal MRI findings revealed that in many cases, abnormal MRI findings were found in the lumbar vertebrae, even if there were no obvious symptoms.

Gianluca et al. [22] compared the MRI findings of adult weightlifters without LBP, whose average age was 25, to age-matched adults in the general population. They reported that there were no significant differences in the Pfirrmann degenerative grades, but proteoglycan degradation and findings of disc degeneration were observed in weightlifters on MRI T1p. Hence, many weightlifters were reported to have lumbar disc degeneration without LBP. Although lumbar disc degeneration is nearly ubiquitous among patients with symptomatic LBP, the causal relationship between lumbar disc degeneration and LBP has yet to be confirmed [23,24]. When patients with LBP are examined by MRI, lumbar disc degeneration is commonly found. However, patients with lumbar disc degeneration do not always have LBP [25,26]. In this study, 2 of the 3 cases with LBP were found to have lumbar spondylolysis or disc protrusion. Only one case had LBP with lumbar disc degeneration alone. Hence, the possibility of LBP is unlikely in patients who have lumbar disc degeneration alone.

In generally, the lumbar disc has poor regenerative capabilities due to poor vascularity and disc nutrition. Therefore, lumbar disc degeneration is often irreversible [27], and this can lead to other problems including disc protrusion, extrusion, or spondylolisthesis [28]. Reports about the natural course of lumbar degeneration in children who have growth plates are scarce. However, in a previous study, Tertti MO et al. [29] showed that 26% of children aged 15 years without LBP had degenerative lumbar changes. Furthermore, Kjaur P et al. [30] showed that approximately 1 of 3 children aged 13 years with LBP had degenerative lumbar changes. In our study, 9 out of 12 (75%) participants had degenerative lumbar disc findings at a high rate and the degenerative disc changes were irreversible, despite the participants being younger than those in previous studies.

The participants who currently have no LBP may develop additional findings in the future, such as disc protrusion, extrusion, or spondylolisthesis. These findings may be symptomatic or asymptomatic; hence, continuous follow-up and observation is necessary.

One of the strengths of this study is its prospective design. Moreover, to our knowledge, the potential changes of the lumbar disc in child and adolescent weightlifting athletes have not been previously investigated.

There were some limitations to our study. First, our subject pool was small. This is largely because of the fact that there are few athletes who weightlift during the growth phase. We intend to continue the investigation and increase the number of cases in the future. Although this was a small cohort, a high incidence of MRI anomalies was detected. We believe that the small sample size may have had an impact, but certainly may not be the only explanation. Second, lumbar spondylosis was evaluated by only the signal changes of MRI, and there was a possibility of over-diagnosis. In this study, a spine surgeon and an orthopedic surgeon evaluated the MRIs twice, and hence, the reliability was considered sufficient. Third, the presence of disc protrusion and extrusion could not be confirmed because we did not have the axial plane on MRI. However, these findings can be reliably diagnosed only on a sagittal plane and can be a cause of LBP. Fourth, there was no control group comprising children of similar ages with growth plates to observe the course of natural lumbar degeneration. However, past studies that focused on 15-year-olds reported that the natural occurrence of lumbar degeneration was about 30%. Hence, the degenerative ratio of this study can be considered high. Finally, the relationship between potential changes on MRI and LBP was not evaluated because the 3-year follow up period was short. Continuing investigation in the future may be warranted. Based on the results of this study, children and adolescents who weightlift at the competition level may develop changes in the lumbar vertebrae with or without symptoms. Further studies may be warranted in the future to investigate the relationship between lumbar disc degeneration and symptoms over a longer course and with a larger sample size. Furthermore, to prevent irreversible changes of the intervertebral disc, it is important to study the risk factors causing lumbar disc degeneration. This could include exercise frequency, duration, and intensity, as well as preventive programs. Based on the results of this study, we hope to help prevent injuries associated with weightlifting in the growth phase.

## Conclusion

This prospective 3-year cohort study of 12 child and adolescent weightlifters revealed abnormal lumbar findings in 11 participants at a high rate on MRI examination. Our findings suggest that resistance training at the competition level could potentially cause irreversible changes in the lumbar vertebra without symptoms.
